# Bioprinting Via a Dual-Gel Bioink Based on Poly(Vinyl Alcohol) and Solubilized Extracellular Matrix towards Cartilage Engineering

**DOI:** 10.3390/ijms22083901

**Published:** 2021-04-09

**Authors:** Mohsen Setayeshmehr, Shahzad Hafeez, Clemens van Blitterswijk, Lorenzo Moroni, Carlos Mota, Matthew B. Baker

**Affiliations:** 1 Biomaterials and Tissue Engineering Department, School of Advanced Technologies in Medicine, Isfahan University of Medical Sciences, Isfahan 81746-73461, Iran; setayeshmehr.m@gmail.com; 2 MERLN Institute for Technology Inspired Regenerative Medicine, Complex Tissue Regeneration, Maastricht University, 6229 Maastricht, The Netherlands; s.hafeez@maastrichtuniversity.nl (S.H.); c.vanblitterswijk@maastrichtuniversity.nl (C.v.B.); l.moroni@maastrichtuniversity.nl (L.M.)

**Keywords:** poly(vinyl alcohol), decellularized cartilage matrix, bioprinting, thiol-ene cross-linking

## Abstract

Various hydrogel systems have been developed as biomaterial inks for bioprinting, including natural and synthetic polymers. However, the available biomaterial inks, which allow printability, cell viability, and user-defined customization, remains limited. Incorporation of biological extracellular matrix materials into tunable synthetic polymers can merge the benefits of both systems towards versatile materials for biofabrication. The aim of this study was to develop novel, cell compatible dual-component biomaterial inks and bioinks based on poly(vinyl alcohol) (PVA) and solubilized decellularized cartilage matrix (SDCM) hydrogels that can be utilized for cartilage bioprinting. In a first approach, PVA was modified with amine groups (PVA-A), and mixed with SDCM. The printability of the PVA-A/SDCM formulations cross-linked by genipin was evaluated. On the second approach, the PVA was functionalized with cis-5-norbornene-endo-2,3-dicarboxylic anhydride (PVA-Nb) to allow an ultrafast light-curing thiol-ene cross-linking. Comprehensive experiments were conducted to evaluate the influence of the SDCM ratio in mechanical properties, water uptake, swelling, cell viability, and printability of the PVA-based formulations. The studies performed with the PVA-A/SDCM formulations cross-linked by genipin showed printability, but poor shape retention due to slow cross-linking kinetics. On the other hand, the PVA-Nb/SDCM showed good printability. The results showed that incorporation of SDCM into PVA-Nb reduces the compression modulus, enhance cell viability, and bioprintability and modulate the swelling ratio of the resulted hydrogels. Results indicated that PVA-Nb hydrogels containing SDCM could be considered as versatile bioinks for cartilage bioprinting.

## 1. Introduction

Additive manufacturing techniques are increasingly used for biofabrication of three-dimensional (3D) scaffolds and constructs in tissue engineering (TE) [1]. Bioprinting offers controlled patterning and deposition of polymeric hydrogels or composites to fabricate well-defined constructs with the capability to combine various material and their compositions [2]. The bioprinting of cell-laden biomaterials, termed bioinks, allows the deposition of cells encapsulated in a defined 3D construct and can develop into living tissue-engineered constructs [3]. However, the available biomaterial inks and bioinks, which balance tailorability with desired performance for bioprinting and tissue growth, remains low [4].

A number of bioprinters with different dispensing principles have been commercialized, including inkjet or droplet-based, extrusion or pressure-based, and laser-assisted bioprinting [5]. All of these systems allow spatial control over bioink deposition while offering distinctive ranges of processable inks formulations. Extrusion bioprinting [6] is one of the most frequently used systems owing to its ease of processing, low cost of adoption, and ability to bioprint high cell densities. Extrusion systems are normally used to dispense in a layer-by-layer fashion filaments in a controlled way and the viscosity and gelation kinetics of the selected bioinks plays a key role in defining their performance [7]. High viscosity formulations normally offer structures with high shape fidelity, but upon gelation, high network density limits encapsulated cells in both mobility and the ability to reorganize the matrix. On the contrary, lower viscosity materials provide a less compact network and more permissive environment for cells but can suffer from low printability or structural integrity [7].

There are many efforts toward bridging this incompatibility gap, where systems not only meet the demands for good printability but also provide a suitable environment for cells [8]. Various hydrogel systems have been developed as biomaterial inks for bioprinting [9], including natural polymers [10] such as gelatin [11], collagen [12], alginate [13], and extracellular matrix (ECM)-derived materials [14] or synthetic polymers such as poly(ethylene glycol) [15], Pluronic [16], poly(vinylpyrrolidone) [17], and poly(vinyl alcohol) (PVA) [18]. Depending on the choice of polymer, different mechanisms can be employed for the cross-linking reactions. Photo-polymerizable systems have recently become a popular choice due to spatial and temporal control of cross-linking [19]. Free radical polymerization of acrylate derivatives has been extensively used in the design of photoreactive bioinks [2]; however, this free-radical chain-growth reaction characteristically yields all carbon kinetic-chain backbones, which greatly affect and tunability of mechanical properties. Photoinitiated thiol-ene cross-linking is well-matched as an alternative cross-linking mechanism and recently has reached utility in biofabrication [10,13,20,21]. The thiol-ene photo-cross-linking can be stoichiometrically controlled, chemospecific, and less sensitive to oxygen and is able to form homogeneous hydrogel networks when compared to free radical acrylate derivatives [22]. In addition, the same thiol-ene chemistry can allow the decoration of the hydrogel scaffold with numerous thiol-incorporating peptides and biomolecules, and dithiol (DT) cross-linkers can be designed to facilitate degradation. An example of thiol-ene chemistry for fabrication of cell-laden hydrogels include hyaluronic acid systems functionalized by both methacrylates and norbornenes to create dual cross-linking systems that allow bioprinting via cross-linking upon exposure to UV [23]. These tools facilitate the creation of uniform constructs with features not achievable with other techniques [13].

PVA is a water-soluble, odorless, tasteless, white polymer widely used in industry [18]. PVA is commonly considered as nontoxic polymer and has found use as an additive for food, cosmetic products, and in food packaging [24], but also for pharmaceutical and biomedical applications [25]. An additional advantage of PVA is the opportunity of post-polymerization modification due to its secondary hydroxyl groups, often accomplished via the formation of esters, ethers, and acetals [26]. Previously, PVA hydrogel scaffolds have been created by different cross-linking methods, via incorporation of polymerizable functionalities, whereas photo-cross-linking is of outstanding attention enabling in situ hydrogel formation [27,28,29,30]. Photo-reactive PVA had been investigated for usage as TE scaffolds, e.g., for in situ polymerization for minimally invasive implantation methods [27]. Furthermore, PVA is modified with allyl succinic anhydride and cis-5-norbornene-endo-2,3-dicarboxylic anhydride to produce macromers with reactive ene groups. The obtained macromers were photo-cross-linked via thiol-ene chemistry (via a thiol modified PVA) and resulted in mechanically tunable hydrogel formulations. Cell studies demonstrated that the resulted hydrogels exhibit low toxicity [18]. PVA hydrogels have been reported to resist protein adsorption and cell adhesion, allowing the incorporation of bioactivity for rationally tuning cell–matrix interactions [31].

ECM materials can either be harvested from cell-derived matrices from in vitro culture or can be obtained directly from native tissue. ECM from either source has to be decellularized to eliminate nucleic acids and cellular components, which may have the potential to cause adverse immunological reactions [32,33]. Some research groups have already established that decellularized cartilage has chondroinductive potential [34]. We recently reported the chondroinductive potential of genipin cross-linked PVA/devitalized cartilage (DC) matrix in vitro, where we observed chondroinductivity of human adipose-derived mesenchymal stromal cells cultured on ECM-based scaffolds [32,35].

In recent years, use of different ECM derivatives has been examined in culturing a variety of cell types, consisting of the usage of solubilized ECM and re-forming it as a hydrogel [36,37]. A key problem of bioprinting ECM-derived material is the high sensitivity of biomaterial ink concentration and viscosity during the bioprinting process, associated with the nozzle diameter selection, combined with the poor or slow gelation after extrusion [38]. However, by incorporation of cross-linkable synthetic materials into naturally derived ECM, one can modulate and improve the printability of the resultant hydrogel. Previous studies have established biomaterial inks based on PVA macromeres, yet lack of bioactivity is a problematic issue regarding to these synthesized materials [18]. Photoactive PVA hydrogels have been functionalized with the cell-adhesive peptide arginylglycylaspartic acid (RGD) and found to support the attachment and spreading of fibroblasts [27]. As also previously shown, incorporation of DC into PVA can achieve both advantage of ECM derivatives and synthetic polymers to consider as a strong vehicle for TE applications [35]. Incorporation of ECM into PVA hydrogel not only can improve biological characteristics of the resulting bioink but can also modulate the viscosity, which is critical parameter in bioprinting. 

**Figure 1:**
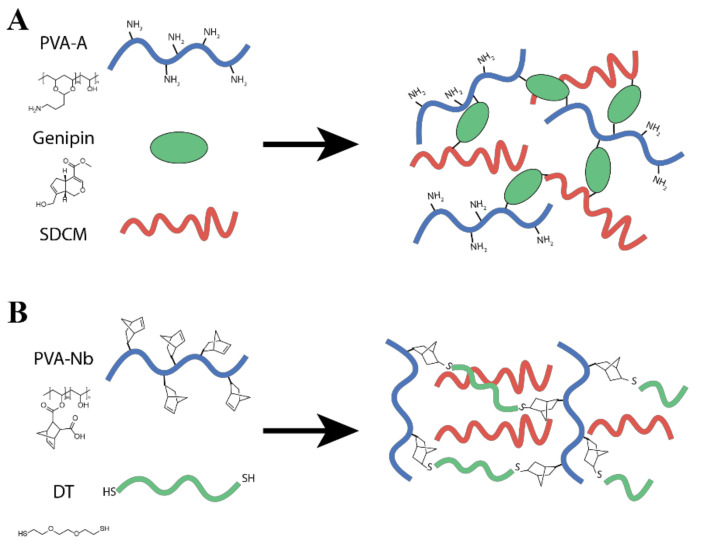
Schematic representations of the (**A**) PVA modified with amine groups (PVA-A)/solubilized decellularized cartilage matrix (SDCM) biomaterial inks formulated from 10 wt% (PVA-A + SDCM) and 0.1% wt/wt genipin and desired PVA-A/SDCM dual-gel. (**B**) PVA functionalized with cis-5-norbornene-endo-2,3-dicarboxylic anhydride (PVA-Nb)/SDCM biomaterial ink.

Hence, the aim of this study was to improve cell compatible and bioactive PVA/ECM bioinks, which can be suitable for cartilage bioprinting purposes. We explored two different formulations (Figure 1) in mixing modified PVA with decellularized ECM. First, amine modified PVA was tested and crosslinked with Genipin (Figure 1A), but poor printing performance was found. We then switched to thiol-ene crosslinking of the PVA (PVA-Nb, Figure 1B) in order to facilitate rapid formation of the multicomponent bioink. These PVA-Nb hydrogels with decellularized ECM were characterized for printing performance, mechanical properties, and the suitability for cartilage tissue engineering.

## 2. Results

### 2.1. Characterization of Cartilage Matrix

**Figure 2:**
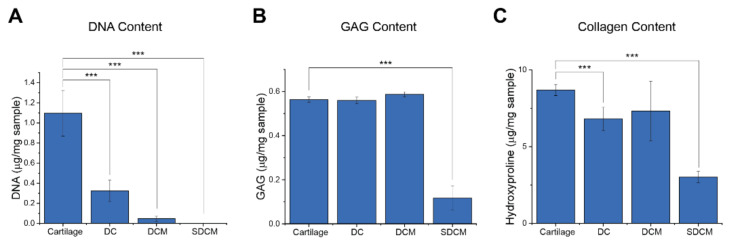
Analysis of key biomolecules during the divitalization process of the processed extracellular matrix (ECM). (**A**) DNA content; (**B**) glycosaminoglycans (GAG) content; and (**C**) collagen content of lyophilized cartilage, devitalized cartilage (DC), decellularized cartilage matrix (DCM), and solubilized decellularized cartilage matrix (SDCM) (Data presented as mean ± SD; *** *p* < 0.001; *n* = 4).

DNA, sGAG, and Hydroxyproline Content: Biochemical content analysis was performed on natural, devitalized, acellular, and solubilized cartilage matrices. The DNA, sGAG, and hydroxyproline contents of natural cartilage were determined to be 1.10 ± 0.20 μg, 6.97 ± 0.20 μg, and 8.70 ± 0.10 μg, respectively (Figure 2). Following devitalization and cryogrinding, there was a 67% reduction in DNA, negligible reduction in GAG, and an 18% reduction in hydroxyproline (*p* < 0.05). Following decellularization, there was a 95.5% reduction in DNA, negligible reduction in GAG, and a 19% reduction in hydroxyproline (*p* < 0.05) (Figure 2). After solubilizing and dialysis, the DNA content further reduced to 0.2% of that of the original lyophilized cartilage (*p* < 0.05). Although there were no significant reductions in GAG content through the devitalization and decellularization, after solubilizing and dialysis, the GAG content further reduced to 12% of that of the natural cartilage. Furthermore, following solubilization and dialysis, the hydroxyproline content was reduced by 35% compared to natural cartilage (*p* < 0.05).

### 2.2. PVA Modification

#### Synthesis of Amine-Functionalized PVA

**Figure 3:**
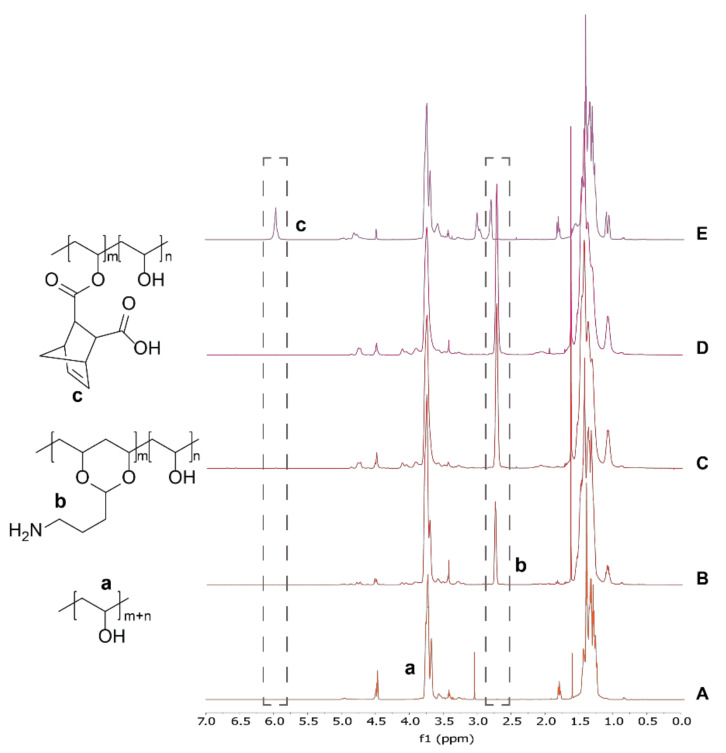
Representative ^1^H NMR spectra of modified polymers used in this study. (**A**–**D**) ^1^H NMR spectrum of different PVA amination for 24 h reaction time point (sample nomenclature PVA-AX-Y, where X corresponds to the reaction time (h) and Y to the maximum theoretical amination percentage). (**A**) Pure PVA, (**B**) PVA-A24-10, (**C**) PVA-A24-50, and (**D**) PVA-A24-100. Successful backbone modification was verified by the peak at 2.9 ppm corresponding to amine linker. (**E**) ^1^H NMR spectrum of norbornene-modified PVA. Successful backbone modification was verified by vinyl peak at 6.25 ppm.

PVA was modified with 4-ABA under acidic conditions to target 10%, 50%, and 100% amination. After reaction and purification, ^1^H NMR showed characteristic peaks near 2.9 ppm, confirming successful amination (Figure 3). The efficiency and amination percentage (relative to polymer subunits) were calculated from the ratio of the alpha methylene peak (2.9 ppm) to the -CH from the polymer backbone (3.9 ppm) in the spectra (Figure 3A–D). The result of different reaction lengths and equivalents of 4-ABA were briefly studied, with the results presented in Table 1. We observed that increasing the reaction time to 24 h led to the highest efficiency for attachment of the 4-ABA to PVA and that attempts at higher functional densities (i.e., 50% and 100%) led to lower functionalization efficiencies—up to 37% functionalization could be obtained in this study.

The cross-linking of the different PVA-A polymers using genipin (0.1% wt/wt) was investigated to evaluate the effect of amination percentage on gelation time (Table 1). The presence of pale blue color and a positive vial inversion test were considered to evaluate gelation time of the hydrogels at 37 °C. As expected, higher functional densities of amines grafted to the PVA resulted in faster gelation kinetics (Table 1). Based on the gelation kinetics, functionalization amount, and functionalization efficiency, PVA with 30% amine modification (PVA-A24-50) and 0.1% wt/wt genipin were chosen to carry forward for 3D bioprinting (referred hereafter as PVA-A).

### 2.3. 3D Bioprinting of PVA-A/SDCM

Different biomaterial inks were formulated from 10 wt% (PVA-A + SDCM) and 0.1% wt/wt genipin to evaluate the printability of the synthesized materials and their suitability for bioprinting approaches. Three different compositions were prepared consisting of: (A) PVA-A, (B) PVA-A/SDCM30, and (C) PVA-A/SDCM50. Due to the low kinetics of the genipin cross-linking, suitable printability was only possible after 2 h once the cross-linking reaction started. After allowing the formulations to pre-cross-link, 3D hydrogels scaffolds were manufactured. Semi porous structures were observed in the PVA-A/SDCM30 and PVA-A/SDCM50 scaffolds (Figure S1). Of note, the printing conditions used to produce these scaffolds match those empirically optimized for best structure. Finally, 5 mm/s deposition speed and 30 kPa extrusion pressure were used to fabricate structures of the three biomaterial ink formulations, leading to poor feature reconstruction for the as-printed scaffold (Figure S1). These scaffolds have shown stability in PBS for over 1 month.

### 2.4. Synthesis of Norbornene-Functionalized PVA

PVA was modified using a norbornene anhydride under acidic conditions to obtain the norbornene ester-modified polymer (Figure 1). Peaks observed in the ^1^H NMR spectrum at chemical shifts of: 6.22 (a, 2H, CH_5_CH), 4.02 (1H, polym-CH-O), 3.28 and 3.09 (2H, CH[ring]), and 1.39 (2H, CH_2_[ring]) confirmed successful norbornene modification [18]. According to the ^1^H NMR spectrum, the calculated norbornene modification was 6.5 mol% with respect to monomer units of PVA (Figure 3E).

### 2.5. Volume Change Analysis and Swelling

**Figure 4:**
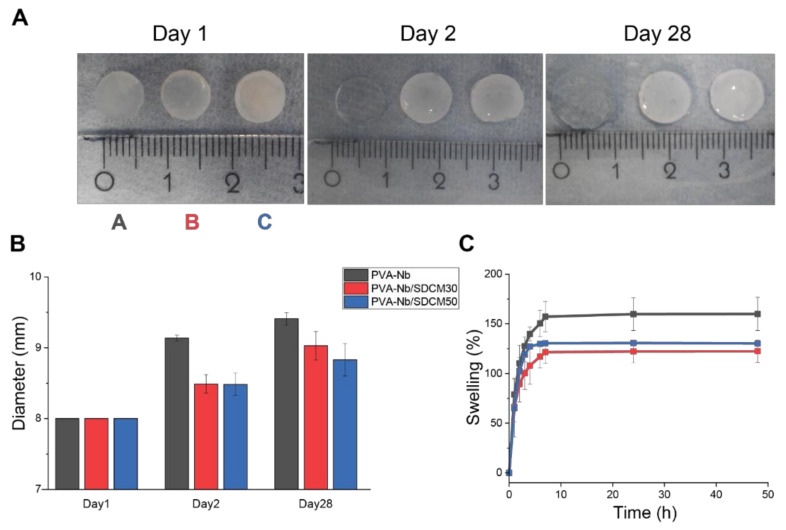
Swelling of norbornene-based hydrogels in phosphate-buffered saline (PBS). (**A**) The photograph of the hydrogels 0, 2, and 28 days after cross-linking and soaking in PBS at 37 °C. Morphology of PVA-Nb hydrogel (10 % wt/vol) cross-linked with 0.5 equivalent of thiol 2,2′-(Ethylenedioxy)diethanethiol (DT) and 2 mM lithium phenyl-2,4,6-trimethylbenzoylphosphinate (LAP). (**A**) PVA-Nb, (**B**) PVA-Nb/SDCM30, and (**C**) PVA-Nb/SDCM50 were successfully cross-linked into hydrogels. (**B**) The sizes of the hydrogels over the course of the 28-day experiment and the (**C**) swelling percentages are presented over the course of 48 h incubation in PBS.

The effect of ECM on the dimensional and structural stability of the composite hydrogels was evaluated, as these are the critical factors in the cartilage healing process. Hence, to evaluate the effect of incorporation of SDCM to PVA-Nb on dimensional stability, different PVA-Nb/SDCM formulations were cross-linked using 0.5 equivalent DT (equimolar functional groups) and LAP into cylindrical (bulk) hydrogels. Photographs of the hydrogels at 0, 2, and 28 days following cross-linking and soaking in PBS at 37 °C are shown in Figure 4A. The results of the diameter measurement showed that inclusion of SDCM to hydrogels decreased the amount of swelling. The average diameter of the PVA-Nb samples showed an increase in day 2 and 28 compared to day 1, which was 9.14 ± 0.04, 9.41 ± 0.09, and 8.00 mm, respectively. While, the average diameter of the PVA-Nb/SDCM50 samples showed a less pronounced increase in day 2 and 28 compared to day 1, which was 8.48 ± 0.16, 8.83 ± 0.23, and 8.00 mm, respectively (Figure 4B). The results of the swelling test during 48 h showed that the PVA-Nb group swelled significantly (168%) more than PVA-Nb/SDCM30 (122%) and PVA-Nb/SDCM50 (130%) groups (Figure 4C).

### 2.6. Mechanical Testing of Cross-Linked Hydrogels

**Figure 5:**
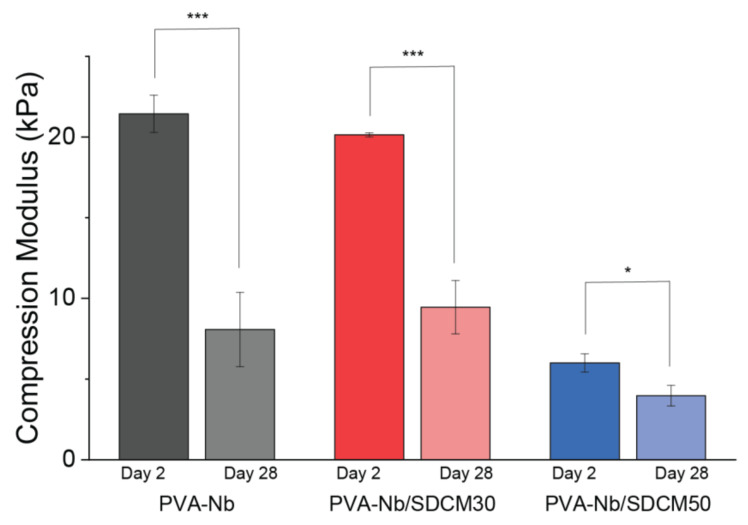
Compression modulus of PVA-Nb hydrogels (10 % wt/vol) cross-linked with 0.5 equivalent DT and 2 mM LAP. PVA-Nb, PVA-Nb/SDCM30, and PVA-Nb/SDCM50 after 2- and 28-days’ incubation in PBS. * *p* < 0.5, *** *p* < 0.01.

The Young’s modulus under compression after incubation in PBS was measured for the different bulk hydrogels after 2 and 28 days (Figure 5). A decrease in compressive mechanical properties was observed with the incorporation of larger amounts of SDCM in the formulation (50%), while the pure PVA-Nb gels and the 30% SDCM formulations had similar mechanical properties both at day 2 and 28. Two days after cross-linking, the compressive modulus of the PVA-Nb was 21.43 ± 1.16 kPa, whereas that of the PVA-Nb/SDCM30 and PVA-Nb/SDCM50 groups were 6% and 72% smaller, respectively (*p* < 0.05) (Figure 5). Furthermore, the compressive modulus of the PVA-Nb/SDCM30 group was 70% higher than that of the PVA-Nb/SDCM50 group (*p* < 0.05). Four weeks after incubation in PBS at 37 °C, the compressive modulus of the PVA-Nb was 8.07 ± 2.30 kPa, which was 62% smaller compared to day 2. Similarly, the PVA-Nb/SDCM30 group showed 53% reduction, while the PVA-Nb/SDCM50 group exhibited 30% reduction in elastic modulus compared to day 2 (*p* < 0.05). Over the 4 weeks of incubation, the PVA-Nb/SDCM50 showed the smallest reduction in mechanical properties (Figure 5).

### 2.7. Live/Dead Assay of Bulk Hydrogels

To evaluate the cytocompatibility of the hydrogels, ATDC5 viability in the bulk hydrogels was investigated at day 1 and 7 via Live/Dead staining (Figure S2). All hydrogel formulations showed over 70% cell viability at all time points measured. Both the PVA-Nb and the PVA-Nb/SDCM30 showed higher numbers of viable cells in day 1 (82% and 88%, respectively) with a slight decrease in the percentage of viable cells over the 7-day experiment (71% and 82%, respectively). The PVA-Nb/DSCM50 showed the lowest day 1 viability of the three samples (70%), yet the percentage of viable cells increased over the 7-day experiment (78%).

### 2.8. Morphology and Distribution of the Chondrocytes

To investigate cell remodeling and cell distribution of cell encapsulated hydrogels, Alcian blue staining was performed. Seven days after encapsulation the ATDC5 cells, hydrogels were stained with Alcian blue and Fast red. A round morphology, demonstrating a central core surrounded by a basophil transition zone stained with Alcian blue was observed (Figure S2-ΙΙ). One of the main challenges in bioprinting is cell sedimentation and ensuring a homogenous distribution of cells within a printed hydrogel [39]; we observed homogeneously dispersed chondrocytes in all hydrogels at day 7.

### 2.9. 3D Bioprinting of PVA-Nb/SDCM

Biomaterial inks were formulated from 10 wt% (PVA-Nb + SDCM), 2 mM LAP, and 0.5 equivalent DT. Three different compositions were prepared consisting: (A) PVA-Nb, (B) PVA-Nb/SDCM30, and (C) PVA-Nb/SDCM50. Scaffolds printed in the geometry of a cube. Porous-like structures can be seen in the PVA-Nb/SDCM50 scaffold (Figure S3C), but not in the printed PVA-Nb construct (Figure S3A). Of note, the printing conditions used to produce these scaffolds match those optimized for best structure. Finally, 5 mm/s deposition speed and 30 kPa extrusion pressure were used to produce constructs of the three biomaterial ink formulations.

### 2.10. Bioink Bioprinting

**Figure 6:**
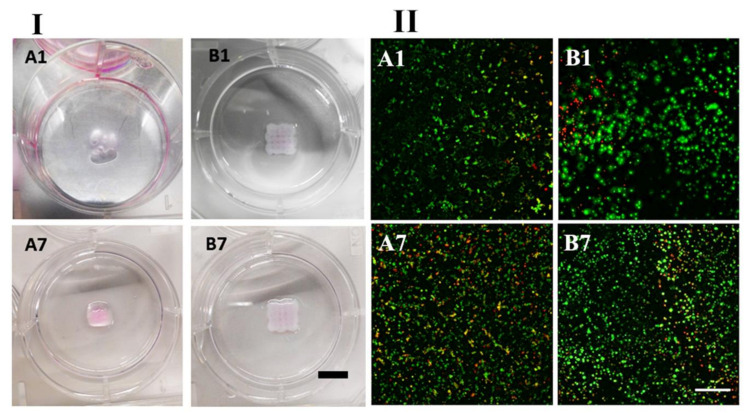
(**І**) Images of 3D bioprinted hydrogels, and (**ІІ**) cell viability in bioprinted constructs. (**A**) PVA-Nb and (**B**) PVA-Nb/SDCM50 at day 1 and day 7. Scaffolds bioprinted in the geometry of a cube. Porous-like structures can be seen in the PVA-Nb/SDCM50 scaffold (scale bar 10: mm). Green stain represents live cells and red stain represents dead cells (scale bar 100 µm).

A syringe cartridge was loaded with PVA-Nb, SDCM, 2 mM LAP, and 0.5 equivalent DT and ATDC5 (1 × 10^7^ cells/mL) in order to make the bioink formulation. Bioinks of two different formulations were tested (A) PVA-Nb and (B) PVA-Nb/SDCM50. Porous scaffolds were bioprinted in the geometry of a cube. Porous-like structures can be seen in the PVA-Nb/SDCM50 scaffold (Figure 6B1). Scaffolds were bioprinted with empirically optimized valued to obtain the best structure to match the theoretical dimensions (side length of 10 mm, 5 strands, 1.50 mm between strands, total height of 4 mm, 160 µm/layer, 25 layers). A 5 mm/s deposition speed with 30 kPa extrusion pressure was used to produce structures of the two bioink formulations.

Employing optimized conditions, multilayer constructions like a simple cubic structure were created with sufficient integrity over 25 layers of bioprinting. As shown in Figure 6, the structural integrity and initial 3D scaffolds of PVA-Nb/SDCM50 structure were well-preserved during culture. Some swelling slightly induced a decrease in the porosity of the constructs, yet porous-like structures were maintained and observed in the X-Y and Z planes as shown in Figure 6-B1,B7. The obtained scaffolds for the PVA-Nb-based bioinks showed limited resolution without a defined porous network. Furthermore, the swelling of this formulation was more pronounced when kept in culture during 7 days.

### 2.11. Live/Dead Assay of Bioprinted Hydrogels

To evaluate the cytocompatibility of the bioink and the bioprinting procedure, bioinks containing ATDC5 were bioprinted into 6-well plates and constructs were cultured in media for 1 week (Figure 6-Ι; A,B). To guarantee that the observed cytocompatibility was well-maintained within all depths of the printed constructs, Z-stack images for each sample were analyzed (Figure 6-ΙΙ; A,B) and quantified (Figure S4). Florescent images of PVA-Nb constructs stained with Live/Dead assays exhibited high cell viabilities (~85%) at day 1, with a slight decrease in cell viability (~75%) at day 7 after printing (Figure 6-ΙΙ; A1,A7). However, the PVA-NB/SDCM50 bioink showed suitable cell viabilities (~60%) at day 1 with a slight increase (~75%) in viability on day 7 (Figure 6-ΙΙ; B1,B7). The results indicated that the viability increased in time in the PVA-NB/SDCM50 inks, while the PVA-Nb inks did not support an increase in viable cells. However, slightly more dead cells could be seen at day 1 in the PVA-Nb/SDCM50 scaffolds as compared to PVA-Nb (Figure 6-ΙΙ; A1,B1).

## 3. Discussion

The aim of this study was to create a PVA/ECM formulation that can be utilized for bioprinting of cartilage constructs. By blending the bioactivity of solubilized and decellularized ECM with the tailorability and control of cross-linking of synthetically modified PVA, benefits from both biomaterials can be combined. In the present study, two approaches were investigated. First, PVA-A biomaterial ink containing SDCM and cross-linked with genipin was investigated, building upon previous promising results with this material as a chondroinductive scaffold [35]. However, the slow cross-linking limited the use of the formulations investigated as biomaterials inks or bioink for bioprinting. An alternative formulation, based on a norbornene-modified PVA (PVA-Nb) was developed in order to improve the gelation process with a rapid photo-induced thiol-ene cross-linking. PVA-Nb formulated with SDCM allowed to obtain bioprint 3D structures, and was proven suitable for bioink formulation showing high cell viability post-bioprinting up to 7 days. In a recently published study, a new class of cartilage ECM (cECM)-functionalized alginate bioink for the bioprinting of cartilaginous tissues was also investigated. The developed bioinks were 3D-printable, supported mesenchymal stromal cells viability post-printing and robust chondrogenesis in vitro in bioinks containing the higher percentage of cECM [40], in line with the similar findings in our current study.

The dense structure of cartilage presents exclusive challenges for decellularization in terms of tolerable elimination of cellular fragments while preserving non-collagenous components [41]. The most common chemicals to remove cell debris from the cartilage consists of SDS, Triton, trypsin, pepsin, hydrochloric acid, and guanidine hydrochloride, however, GAG loss during decellularization is known to occur using these materials [42]. Furthermore, retaining the GAG content through decellularization could potentially improve DCM properties including the elasticity, tensile, and compressive properties of DCM [43], as well as chondroinductivity effects in vitro and in vivo [44]. In a previous study, decellularization of bovine nucleus pulposus tissue was investigated using sodium deoxycholate, SDS, and Triton X-100, in combination with freeze-thaw cycles and DNase treatment [45]. The results showed no changes in collagen II maintenance, but there was significant GAG loss (80%) compared to the native cartilage. In another study, using the various detergents (Triton X-100, sulfobetaine-10, and sulfobetaine-16) in short cyclic intervals resulted in 49–55% GAG retention, while effectively removing cellular components [46]. In the present study, DC was decellularized using a modified method previously established via osmotic shock, detergent washes, and enzymatic treatment to preserve GAG and collagen content as much as possible (14). By documenting the biochemical makeup of the material during the decellularization process, it was observed that large decreases of DNA (95% removed) occurred during the decellularization process with good retention of GAGs (~100%) and hydroxyproline (82%) content. For consistency, both of our attempted approaches used identical DCM.

Various decellularized ECM-derived bioinks, such as liver, muscle, tendon, cardiac tissue, and cartilage, have been developed, which are used ECM in solubilized form [42]. The solubilization process occurred after lyophilization and destruction via pepsin digestion in the presence of acetic or hydrochloric acid solution following neutralization of the pH to 7.4. The resulted pregel was appropriate for combining with cells [47]. However, to use the DCM as a bioink, the decellularization process was followed by a solubilization and purification process via dialysis, which led to further decreases in DNA content (not detected), yet also decreased the GAG (to 12%) and hydroxyproline content (to 35%).

In our initial approach, PVA was successfully modified with amine functional groups. We observed that the degree of modification could be controlled via stoichiometry or reaction time. A decrease in reaction efficiency was observed when targeting higher levels of amination. Higher levels of functionalization led to faster genipin cross-linking speeds; however, these cross-linking kinetics within the hour range ultimately led to difficult optimization and a limited suitability for bioprinting. A recently developed approach for formulation of bioinks is the pre-cross-linking of precursor solutions to a state of higher viscosity, followed by final cross-linking after bioprinting [48]; a similar approach was employed here. Nevertheless, the 3D bioprinted structures from these formulations were not satisfactory (Figure S2A–C) and were only able to be manufactured after the pre-cross-linking reaction was initiated inside of the cartridge.

Noticing the poor cross-linking kinetics associated with genipin, in our second approach, we turned to the photoinitiated thiol-ene reaction chosen to introduce fast cross-linking in the 3D structure. Norbornene is one of the most reactive substrates for the radical thiol-ene reaction. PVA was successfully modified with cis-5-norbornene-endo-2,3-dicarboxylic anhydride (PVA-Nb, 7% functionalization). Different compositions of the PVA and SDCM were prepared with consistent solids content (10 wt%) and a varying composition (0%, 30%, and 50% SDCM). These hydrogel formulations showed rapid gelation upon exposure to UV light and were deemed promising for use as a bioink. Rathan et al. showed that alginate-SDCM bioinks containing 0.2% and 0.4% (wt/vol) SDCM and 2.45% alginate in DMEM had similar behavior to the formulations developed in this study in relation to the increase in resolution with the increase in SDCM and the overall cell viability observed [40].

Previous studies on formulation of bioinks based on solubilized ECM demonstrated that by manipulating the concentrations, molecular weights, and geometries, a range of shear elastic modulus values could be reached, spanning from 113.66 Pa to 19.798 ± 0.24 kPa, near 200-fold increase in stiffness [49]. In addition, the value of Young’s modulus of pericellular matrix around chondrocytes reported in previous studies is in the order to 10–100 kPa [50]. Furthermore, the elastic modulus of healthy articular cartilage is in the range from 130 to 573 kPa [51]. However, comprehensive experiments with PVA/SDCM were conducted to evaluate the influence of the SDCM ratio in water uptake/swelling, mechanical properties, and bioprintability of the different formulations. The results showed that the mass swelling ratio decreased with increasing of SDCM content. Compression testing showed that both the pure PVA-Nb and the SDCM30 had similar mechanical properties over time (up to day 28 in PBS). Inclusion of higher amounts of SDCM (50%) led to a decrease in mechanical properties. These studies suggest that there is a delicate relationship between composition and cross-link density. As the PVA fraction is decreased (thereby decreasing the covalent cross-link density), SDCM can reinforce the hydrogel to a point; however, adding larger amounts of SDCM to the composition begins to compromise the compressive modulus. All in all, the PVA-Nb/SDCM50 was chosen as a bioink for bioprinting proposes, because of proper printability and higher cell compatibility compared to other formulations.

Pepsin and HCl solutions have been employed for ECM digestion in previous studies. These harsh environments might be predictable to result in undesirable degradation of biochemical content [49]. However, we have demonstrated the efficiency of this solubilizing process approach, and the resulted SDCM has shown to significantly increase ATDC5 cell viability when incorporated into the PVA hydrogels. We observed that the cytocompatibility of PVA-Nb formulations depended on the SDCM concentration with formulations containing 30 and 50 wt% SDCM exhibiting higher cell viability at day 7 compared to no SDCM incorporation. The applicability of the synthesized PVA-Nb/SDCM hydrogels for bioprinting was tested and results showed that this hydrogel exhibited a defined porous structure, which is dependent on the SDCM concentration. The structural integrity and initial 3D geometries of structure of PVA-Nb/SDCM50 hydrogels was well preserved after deposition. Results indicated that PVA-Nb/SDCM hydrogels can be considered as the versatile bioinks for cartilage bioprinting, as further adjustments to the DT cross-linking length and cross-linking density can be explored (via the PVA component) without drastically changing formulation viscosity.

## 4. Materials and Methods

### 4.1. Preparation of Solubilized Decellularized Cartilage

Three calf knees (males that were approximately 6–8 months old) were purchased from a local abattoir (Maastricht, Netherland). Articular cartilage from the knee joints was removed and collected using scalpels. The cartilage was then rinsed twice in PBS and stored at −20 °C. After freezing overnight, the cartilage was thawed (this was repeated three times) and then coarsely powdered with liquid nitrogen using a freeze-mill (SPEX SamplePrep, Metuchen, NJ, USA). This devitalized cartilage (DC) [14] was powdered to improve the diffusion of the solutions used for the decellularization process. The DC was placed into dialysis bags (3500 MWCO) and decellularized using a modified method previously established via osmotic shock, detergent washes, and enzymatic treatment [14]. The DC-containing bags were exposed in a hypertonic salt solution (HSS) under mild stirring overnight at room temperature. The bags were then placed in a stirrer at 220 rpm and washed twice with Triton X-100 (0.01% *v*/*v*) followed with HSS to permeabilize intact cellular membranes. The tissue was then exposed overnight with Benzonase (0.0625 KU mL^−1^) at 37 °C, then, the tissue was further exposed overnight with sodium lauroyl sarcosine (SLS, 1% *v*/*v*), and finally, the tissue was washed with ethanol (40% *v*/*v*) at 50 rpm and then in PBS at 50 rpm followed by 24 h of rinsing with distillated water (dH_2_O) at 220 rpm. The tissue was then removed from the bags, frozen, and lyophilized. Decellularized cartilage matrix (DCM) was solubilized via a modified protocol from a previously described method [14]. DCM powder was first mixed in 0.1 M HCl at a concentration of 10 mg DCM per mL HCl. Pepsin was then added at a concentration of 1 mg/mL and the solution was stirred at 200 rpm for 3 days at room temperature. The solution was then brought back to physiological pH, by adding 1 M NaOH. The solubilized DCM (SDCM) was then centrifuged at 10,000× *g* for 3 min and the supernatant was collected, frozen, lyophilized, cryo-milled (freeze-mill SPEX SamplePrep, Metuchen, NJ, USA) and stored at −20 °C until further use.

### 4.2. Biochemical Analysis

DNA Quantification: Tris-EDTA-buffered solution containing 1 mg/mL Proteinase K, 1 μg/mL iodoacetamide, and 18.5 μg/mL pepstatin A (all materials from Sigma-Aldrich, St. Louis, MO, USA) was used to digest each sample (*n* = 3) at 65 °C for 24 h. The CyQUANT DNA assay kit (Molecular Probes, Eugene, OR, USA) was used to quantify DNA content of samples according to the manufacturer’s instructions, using a spectrofluorometer at 480/520 nm excitation/emission wavelength (CLARIOstar microplate reader; BMG Labtech, Cary, NC, USA) [52,53].

Sulfated Glycosaminoglycans (sGAG) Quantification: sGAG content was measured by following the method previously described on the Proteinase K-digested samples [53,54,55]. Briefly, sGAG content was determined spectrophotometrically with the 9-dimethylmethylene blue chloride (DMMB, Sigma-Aldrich) dye in PBE buffer (3.72 g/L Na_2_EDTA and 14.2 g/L Na_2_HPO4, pH 6.5) using a multiwell plate reader (Bio-TEK Instruments, Winooski, VT, USA) at 520 nm. The sGAG concentration of each sample was obtained by interpolation of its absorbance from the standard curve [53].

Collagen Quantification: The hydroxyproline assay kit (MAK008, Sigma-Aldrich) was used to quantify hydroxyproline content of samples according to the manufacturers’ instructions. For this purpose, 100 µL of 12 M HCl (Sigma-Aldrich) was used to digest 10 mg of lyophilized samples (*n* = 3 for each group) at 120 °C for 3 h [54,55,56]. Finally, the absorbance of the hydroxyproline standard solution and digested samples was measured using multiwell plate reader (Bio-TEK Instruments) at 520 nm. After drawing the standard curve, hydroxyproline concentration of each sample was obtained by interpolation of its absorbance from the standard curve [35].

### 4.3. PVA Modifications

**Figure 7:**
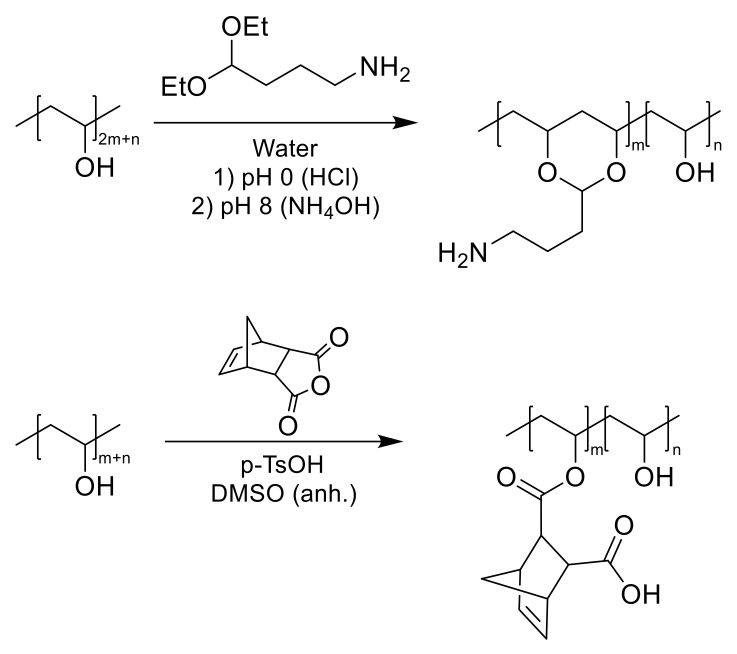
Schematic representation of the modification of poly(vinyl alcohol) (PVA) with amine groups (**top**) and 2-norbornene groups (**bottom**).

Synthesis of Amine-Functionalized PVA: PVA was modified with primary amine functional groups via a method previously described (Figure 7) [35,57]. Briefly, PVA was dissolved in dH_2_O at 90 °C to prepare a 12% wt/vol solution. An equal volume of 10 mol% 4-aminobutyraldehyde diethyl acetal (4-ABA) (Sigma-Aldrich) was added dropwise to the solution (PVA was modified with three different target aminations, i.e., 10, 50, and 100 mol%). The pH was decreased close to zero by adding dropwise HCl 12 M, and then, the solution was mixed up to 24 h. Then, the pH was increased to 8.0 by adding an adequate quantity of NH_4_OH in order to quench the reaction. The resulted solutions were purified by dialysis membrane (MWCO 3500, Sigma-Aldrich) in dH_2_O and finally lyophilized. Samples were collected in three time points during reaction (1, 2, and 24 h) for amination analysis. Sample nomenclature was defined as PVA-A*-** where * corresponds to the reaction time (h) and ** the targeted amination percentage. NMRs are shown in Figures S5–S7.

Synthesis of Norbornene-Functionalized PVA: PVA was modified with cis-5-norbornene-endo-2,3-dicarboxylic anhydride (Nb) via a method previously described (Figure 7) [18]. Briefly, a 20% (wt/vol) solution of Nb (0.5 equivalent with respect to PVA subunits) in anhydrous DMSO was added dropwise to a stirred 4% (wt/vol) PVA solution in anhydrous DMSO, and 0.125 wt% p-toluene sulfonic acid (p-TsOH) was added as catalyst at 50 °C under nitrogen atmosphere and stirred during 24 h. After cooling to room temperature, the reaction mixture was dialyzed (MWCO 3500, Sigma-Aldrich) against dH_2_O (7 cycles), sodium carbonate buffer (pH ≈ 8.2, overnight, 1 cycle), and again dH_2_O (7 cycles). The aqueous solution was evaporated and dried in a rotary evaporator, to obtain functionalized PVA-Nb as a white powder. NMRs are shown in Figure S8.

### 4.4. Nuclear Magnetic Resonance (^1^H NMR) Spectroscopy

^1^H NMR was used to confirm the modification of the PVA with amine and norbornene groups. For this, samples were dissolved in D_2_O, and the spectra was acquired using a Bruker 700 spectrometer. The signal of deuterated solvent (D_2_O at 4.79 ppm) was used as reference [13].

### 4.5. PVA-A/SDCM and PVA-Nb/SDCM Hydrogel Fabrication and Characterization

PVA-A/Genipin Gelation Time: To investigate the influence of the amination percentage with the gelation time, PVA-A hydrogels with different degrees of amination were cross-linked with genipin (0.1% dry weight of genipin/dry weight of sample (wt/wt)). The color change to pale blue and vial inversion test were used to show PVA-A cross-linking after 72 h at 37 °C. Briefly, different modified PVA-A formulations were dissolved in dH_2_O at 60 °C, and nine different PVA-A solutions were prepared. In addition, genipin (sc-203057A, Santacruz, Santa Cruz, CA, USA) was prepared in ethanol at the concentration of 0.1% wt/wt. Then, genipin was added to each PVA-A solution and homogenized. Aliquots of homogenized solution (500 µL) were placed in silicon molds and subsequently cross-linked in incubator at 37 °C for 3 days [34].

3D Bioprinting of PVA-A/SDCM Biomaterial Inks: To evaluate printability of the PVA-A/SDCM, different biomaterial inks were formulated from 10 wt% (PVA-A + SDCM) and 0.1% wt/wt genipin (Figure 1). Three different compositions were prepared consisting: (A) PVA-A, (B) PVA-A/SDCM30 (70% PVA-A:30% SDCM), and (C) PVA-A/SDCM50 (50% PVA-A:50% SDCM). The biomaterial inks were loaded into syringes, assembled into a custom holder, designed to hold a cartridge. Bioprinting was carried out with a G15 needle (1.4 mm ID) on a BioScaffolder (GeSiM—Gesellschaft fuür Silizium-Mikrosysteme mbH, Radeberg, Germany) controlled through proprietary software. The hydrogels were printed in the geometry of a cube (10 mm × 10 mm × 2 mm), with a designed side length of 10 mm (3 strands, 3.50 mm between strands), and a total height of 2 mm (80 μm/layer, 25 layers).

PVA-Nb/SDCM Hydrogel Fabrication and Characterization: Due to the step growth of thiol-ene reaction, the selection of cross-linker has a key outcome on the resulted network, such as mechanical properties and mesh size [13]. We chose to use a small bifunctional thiol 2,2′-(Ethylenedioxy)diethanethiol (DT) to cross-link the hydrogel system. Lithium phenyl-2,4,6-trimethylbenzoylphosphinate (LAP) was employed as the photoinitiator since it is efficient at 365 nm, is water-soluble, and has shown low toxicity when used to cross-link cell-laden hydrogels [58]. Stock solutions of the PVA-Nb, SDCM, DT, and the photoinitiator LAP were prepared in phosphate-buffered saline (PBS) and combined to obtain the desired hydrogel formulations (Figure 1). Three formulation consisting of PVA-Nb/SDCM (100:0), PVA-Nb/SDCM30, and PVA-Nb/SDCM50 were prepared. All formulations contained 10 wt% macromere (PVA + SDCM), 0.5 equivalent DT (with respect to Nb subunits), and 2 mM LAP. Adequate volumes of each formulation were pipetted into a PDMS cylindrical molds (well diameter of 8 mm and thickness of 3 mm). The molds were exposed to UV light (50 mW/cm^2^ at 10 cm distance) for 60 s to fabricate bulk hydrogel samples.

### 4.6. Swelling Degree and Volume Changes

The swelling of the hydrogels was measured via the mass swelling ratio to evaluate the density changes over time of the network structure. Bulk hydrogel samples were equilibrated for 48 h and the swollen weight was recorded at different time points. The swelling degree was calculated as the ratio of swollen weight to initial weight [35]. The geometric mean diameter of each gel was measured directly using a caliper to calculate gel dimension on day 1, 2, and 28.

### 4.7. Mechanical Testing of the Bulk Hydrogels

The gels were allowed to swell to equilibrium in PBS and mechanical testing was performed at day 2 and 28. The gels (*n* = 3) were compressed with load cell of 1000 g in an unconfined setup until mechanical failure at a rate of 0.01 mm/s, and the compressive modulus was calculated as the slope of the linear portion of the stress–strain curve (i.e., 5–15% strain) [35].

### 4.8. Cell Culture

A teratocarcinoma-derived chondrogenic cell line (ATDC5) was used for cell study. The cells were cultured at a density of 1 × 10^5^ cells.cm^2^ in DMEM (F-12) media supplemented with 1% penicillin streptavidin and 5% fetal bovine serum. Cells were subculture at 80% confluence [59].

### 4.9. Live/Dead Assay of Hydrogels

The viability of the ATDC5 exposed to the hydrogel environments was evaluated using a LIVE/DEAD viability/cytotoxicity kit (Thermo Fisher, Waltham, MA, USA). Before cell seeding, the materials were sterilized using filtration, freeze drying, and UV irradiation for 2 h. After a week culture, 1 mL of calcein-AM stock solution (1 μM) was added to each scaffold and incubated for 20 min at 37 °C. After that, 1 mL of the ethidium homodimer-1 stock solution (0.036 μM) was added to the wells and incubated for an additional 10 min at 37 °C. The dye solutions were then aspirated from the wells, and 1 mL of phenol red-free medium (DMEM, Sigma-Aldrich) was added to wells before imaging. Calcein–AM, a cell-permeant dye is converted to a green-fluorescent calcein by viable cells, and ethidium homodimer-1 binds to nucleic acids of cells with damaged membranes to produce red fluorescence. For cell imaging, a live cell imaging Nikon TI-E with environmental control with a 20× objective (WD = 15 mm, NA = 0.3) was used. Images were typically acquired via 1024 μm × 1024 μm scans with Z stacks of 5–10 μm on three different hydrogels. Cell viability was estimated through quantification of the number of live cells over total number of cells using ImageJ software [13].

### 4.10. Histological Analyses

Alcian blue staining is usually used at pH = 2.5, in order to be fixed with acidic groups of carboxylic muco-polysaccharides by electrostatic binding. Acidic polysaccharides such as glycosaminoglycans in cartilages will be stained blue, cytoplasm will be stained pale pink, and nuclei will be stained pink to red. Alcian blue staining was performed in cultured constructs to investigate cell remodeling after encapsulation in the hydrogels. Briefly, after a week culture, ATDC5-containing constructs were fixed during 48 h at 4 °C in 10% buffered formalin in PBS solution. Constructs were processed by dehydration, clearing, paraffin embedding, and sectioning. Xylene-cleared sections were treated with Alcian blue 1% for 45 min and then with 0.1% nuclear Fast red for 3 min; rinsed with distilled water; and dehydrated, cleared, and mounted on microscope slides [47].

### 4.11. 3D Bioprinting of the PVA-Nb/SDCM Biomaterial Inks and Bioinks

To evaluate printability of the PVA-Nb/SDCM, biomaterial inks were formulated from 10 wt% (PVA-Nb + SDCM), 2 mM LAP, and 0.5 equivalent DT. Three different compositions were prepared consisting: (A) PVA-Nb, (B) PVA-Nb/SDCM30, and (C) PVA-Nb/SDCM50. To evaluate cell compatibility of the bioprinting approach and synthesized PVA-Nb/SDCM biomaterials inks, PVA-Nb with specific formulations of photoinitiator and cross-linker used as bioinks were prepared with the addition of ATDC5 cells (10 million cells/mL). The biomaterial inks and bioinks were loaded into black syringes, assembled into a custom holder, designed to hold a cartridge and LED light source (Thorlabs, 365 nm, 10 mW/cm^2^). Bioprinting was carried out with a G15 needle (1.4 mm ID) on a BioScaffolder (GeSiM—Gesellschaft fuür Silizium-Mikrosysteme mbH, Germany) controlled through proprietary software. In general, scaffold geometries and settings were set to a cube (10 mm × 10 mm × 2 mm), comprising 5 meandered strands placed at a distance of 2 mm apart. The deposition angle was turned 90° after each layer. Height of each layer was set to 0.2 mm, and the number of layers was varied according to experiment requirements [13].

### 4.12. Statistical Analysis

For the statistical analysis, data were evaluated using the Student’s *t*-test.

## 5. Conclusions

The aim of this study was to develop cell-compatible PVA/SDCM biomaterial inks that can be used for cartilage bioprinting purposes. PVA-based synthetic hydrogels allow for good control of mechanical properties, but suffer from a lack of biological properties. In contrast, ECM components contain structures that are ideal for cell viability. Hence, hybrid hydrogels consisting of PVA and SDCM, combining the advantages of both PVA and ECM derivatives, are suitable candidates for TE applications such as bioprinted constructs for cartilage regeneration. The initial PVA-A system developed in this study will require further cross-linking optimization to allow suitable bioprintability. Conversely, the thiol-ene reaction enabled by the PVA-Nb system allowed a suitable bioprintability that was further enhanced with the addition of SDCM. Different composition of the PVA and SDCM supported a high cell viability after bioprinting in the PVA/SDCM50 formulation. The structural integrity of PVA-Nb/SDCM50 hydrogels was well-preserved during culture. Our results indicated that the mixing of well-defined synthetic polymers like PVA-Nb, can be combined with SDCM in order to create promising bioink formulations.

## Figures and Tables

**Table 1:** Poly(vinyl alcohol) (PVA) amination in different concentrations and different time points. The results of the gelation time of the different PVA modified with amine groups (PVA-A) polymers using genipin (0.1% wt/wt) as a cross-linker.

Sample Name ^a^ PVA-AX-Y	Reaction Time (h)	Target Amination (mol%)	Calculated Amination (mol%)	Efficiency (%)	Gelation Time (h)
PVA-A1-10	1	10	3.7	37	72
PVA-A1-50	1	50	8.8	17.6	72
PVA-A1-100	1	100	18.0	18.0	48
PVA-A2-10	2	10	5.2	52	72
PVA-A2-50	2	50	14.9	29.8	48
PVA-A2-100	2	100	23.7	23.7	24
PVA-A24-10	24	10	9.7	97	72
PVA-A24-50	24	50	29.7	59.3	24
PVA-A24-100	24	100	37.4	37.4	24

^a^ In the sample naming, X corresponds to the reaction time (h) and Y corresponds to the maximum theoretical amination percentage.

## Data Availability

The data presented in this study are available in this article Int. J. Mol. Sci. and its Supplementary Material.

## Supplementary Materials

The following are available online at https://www.mdpi.com/article/10.3390/ijms22083901/s1, Figures S1–S8.
