# Supplementary material for: Bioprinting Via a Dual-Gel Bioink Based on Poly(Vinyl Alcohol) and Solubilized Extracellular Matrix towards Cartilage Engineering
Source: Int J Mol Sci. 2021 Apr 9;22(8):3901. doi: 10.3390/ijms22083901 (PMC8069267; doi:10.3390/ijms22083901)
Supplement: Supplementary file 1 [file ijms-22-03901-s001.pdf]

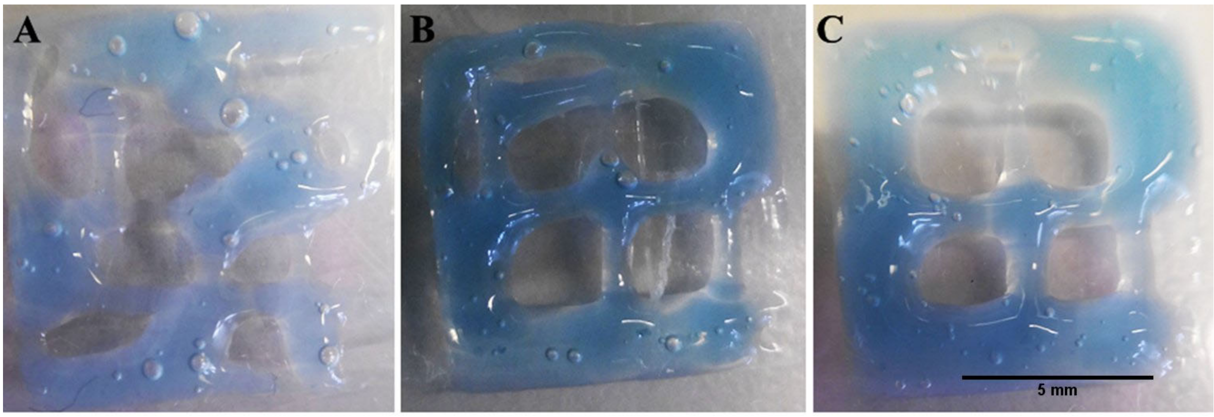

Figure S1. Images of. 3D printed biomaterial inks A) PVA-A, B) PVA-A/SDCM30, and C) PVA-A/SDCM50 crosslinked by 0.1% (wt/wt) genipin as crosslinker (scale bar 5 mm). Scaffolds printed in the geometry of a cube (theoretical side length = 10 mm,(3 strands, 3.50 mm between strands).

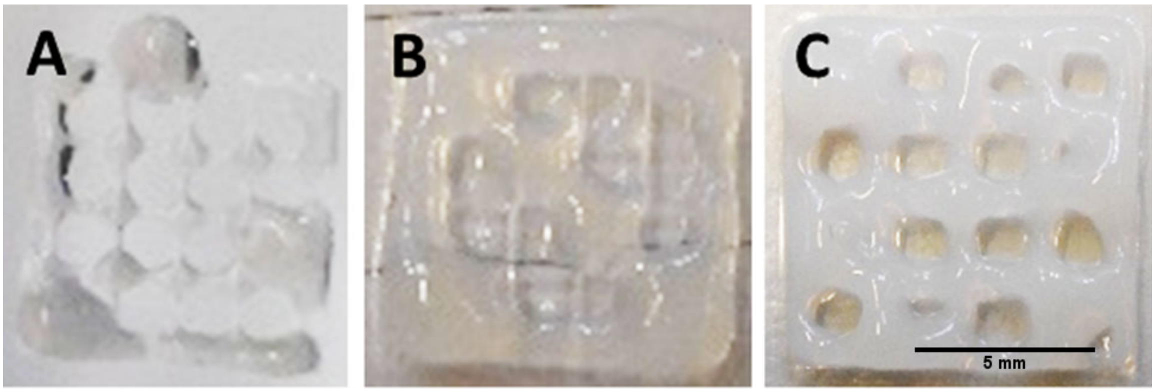

Figure S2. Images of 3D printed hydrogels A) PVA-Nb, B) PVA-Nb/SDCM30, and C) PVA-Nb/SDCM50 (scale bar 5 mm). Scaffolds printed in the geometry of a cube (theoretical side length = 10 mm, 5 strands, 1.50 mm between strands, total height = 2 mm). Porous-like structures can be seen in the PVA-Nb/SDCM50 scaffold. .

I

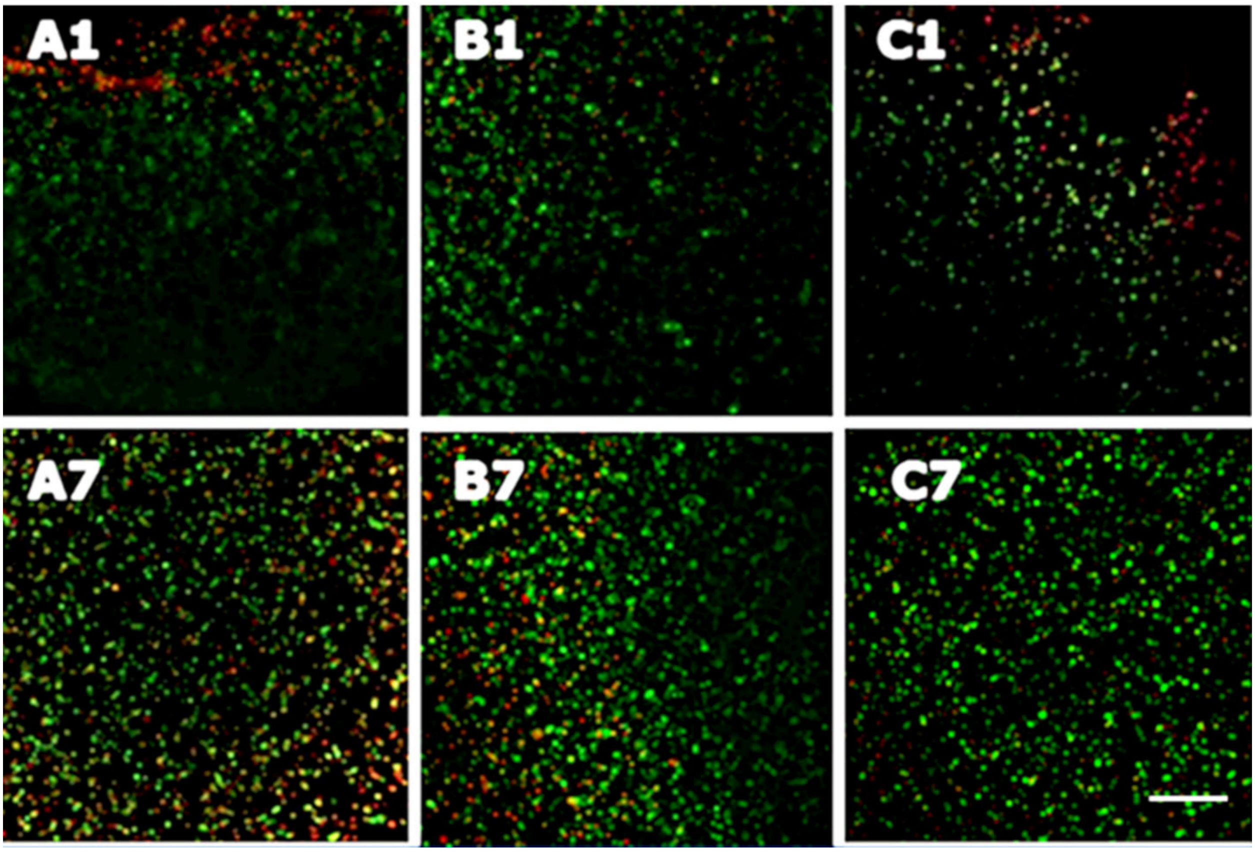

II

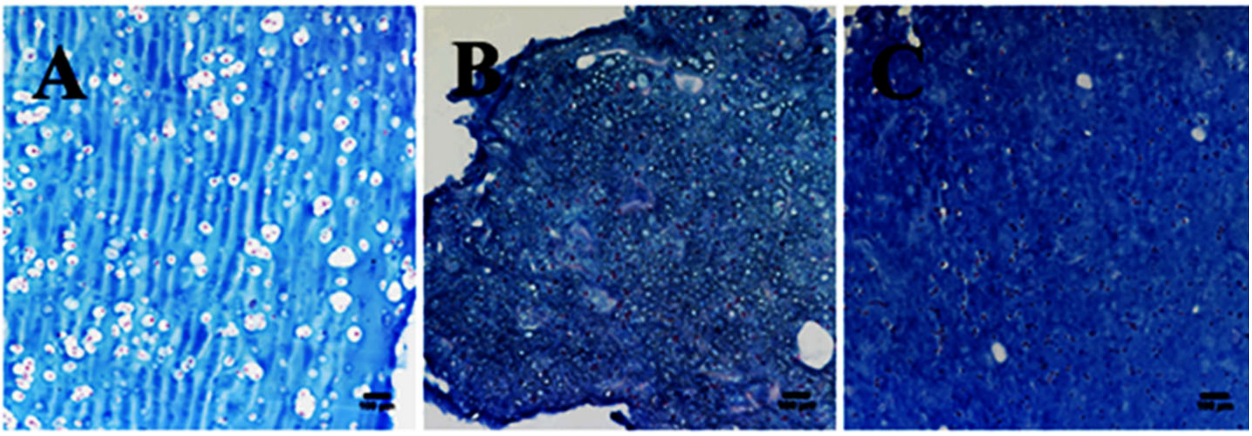

III

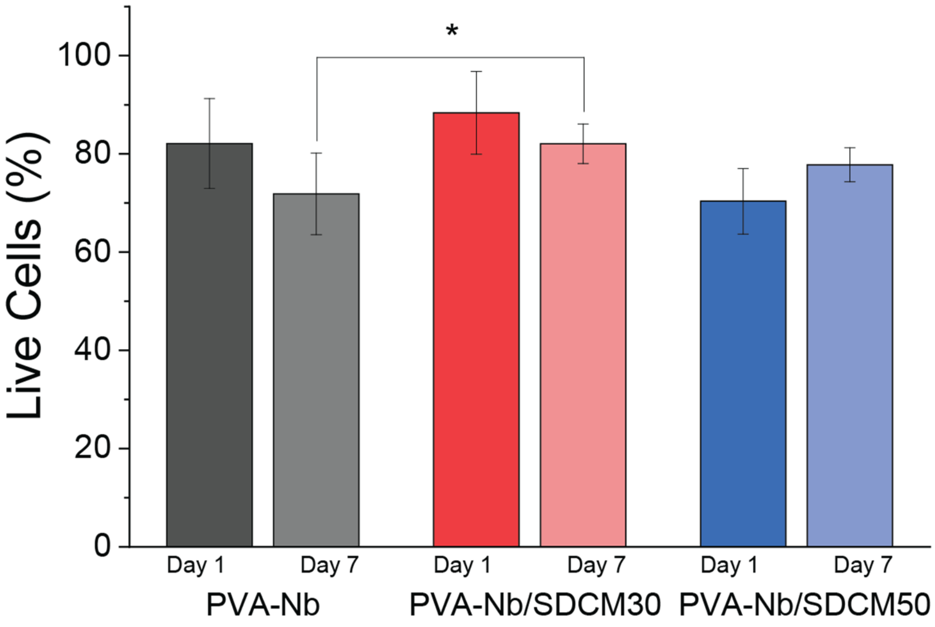

Figure S3. I) Cell viability of ATDC5 cells in the bulk hydrogels, A) PVA-Nb, B) PVA-Nb/ SDCM30 and C) PVA-Nb/ SDCM50 over day 1 and 7. Green stain represents live cells and red stain represents dead cells (scale bar: 100 µm). II) Morphology and distribution of ATDC5 cells in the bulk hydrogels over day 7 using alcian blue – fast red staining (scale bar: 100 µm). III) Quantification of cell viability of the bulk hydrogels after 1 and 7 days of culture. n = 5, *$p < 0.05$.

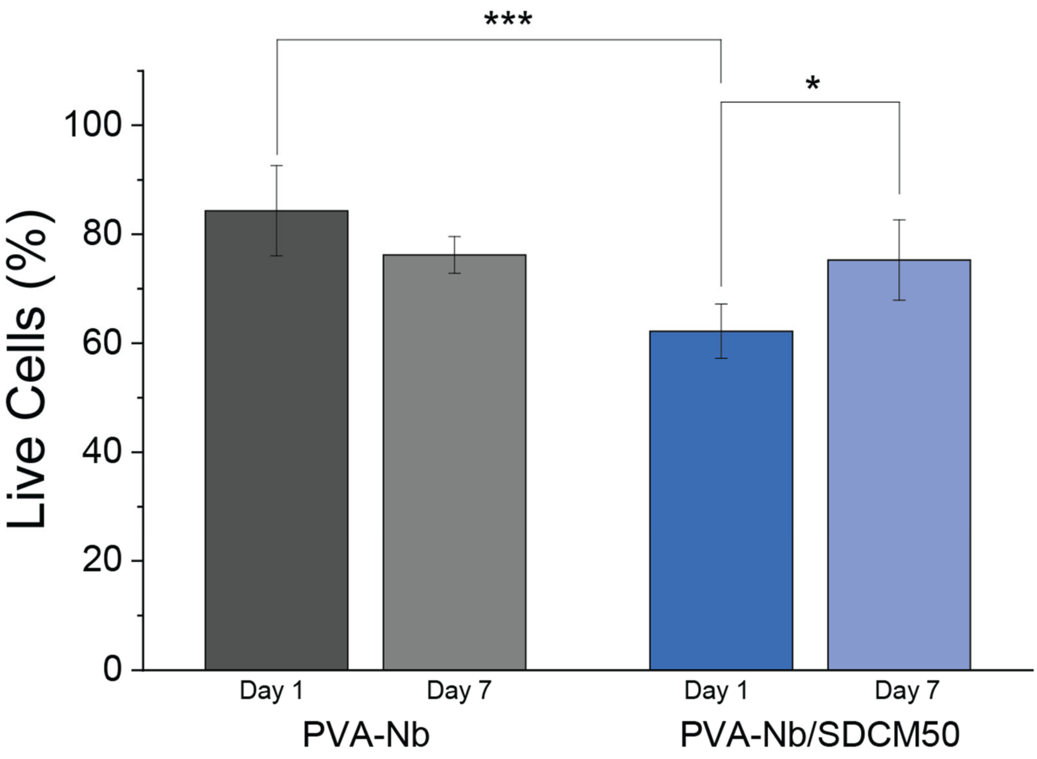

Figure S4. Quantification of cell viability of printed constructs after 1 and 7 days of culture. n = 5, *p < 0.05, ***p<0.01.

MERLN_MS_20170503.1.fid
PVA 1-1, 1D ZGpr, in D2O, 24 scans, T=325K
D1=5s, AQ=1.46s

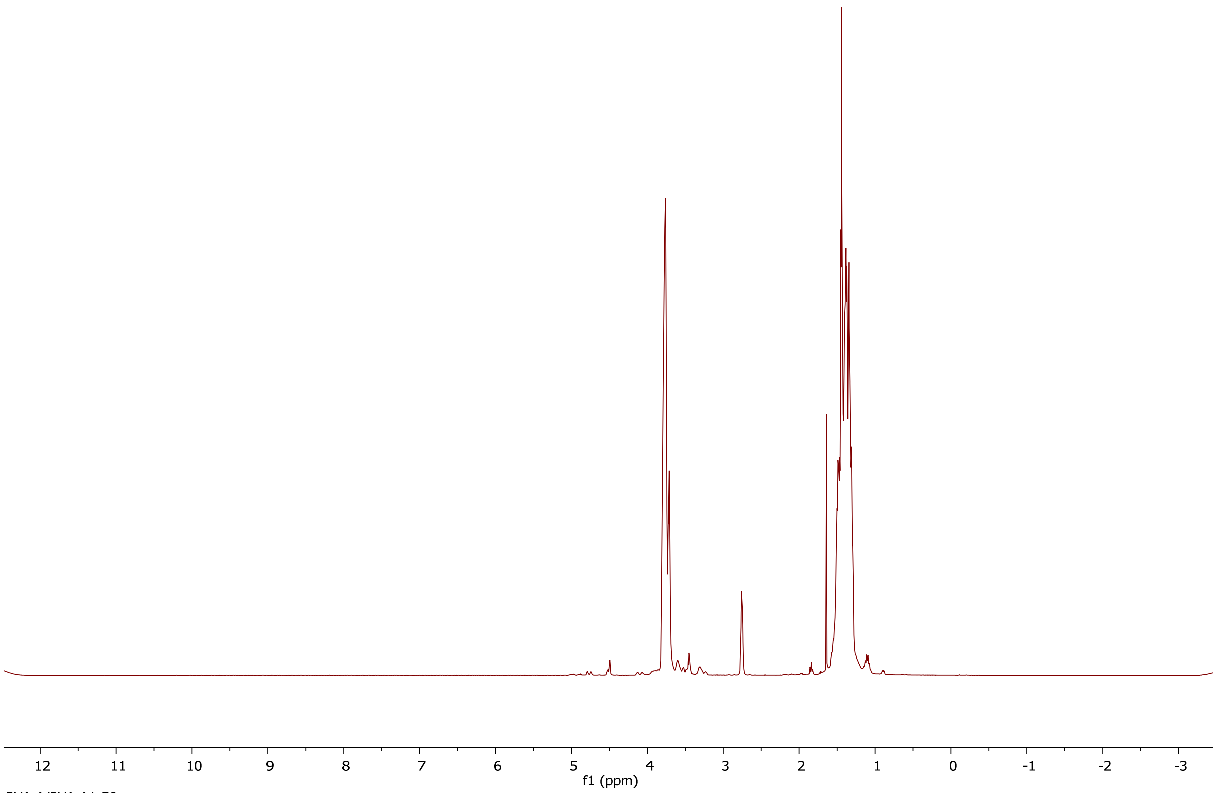

PVA-A/PVA-A1-50
PVA 1-2, 1D ZGpr, in D2O, 24 scans, T=325K
D1=5s, AQ=1.46s

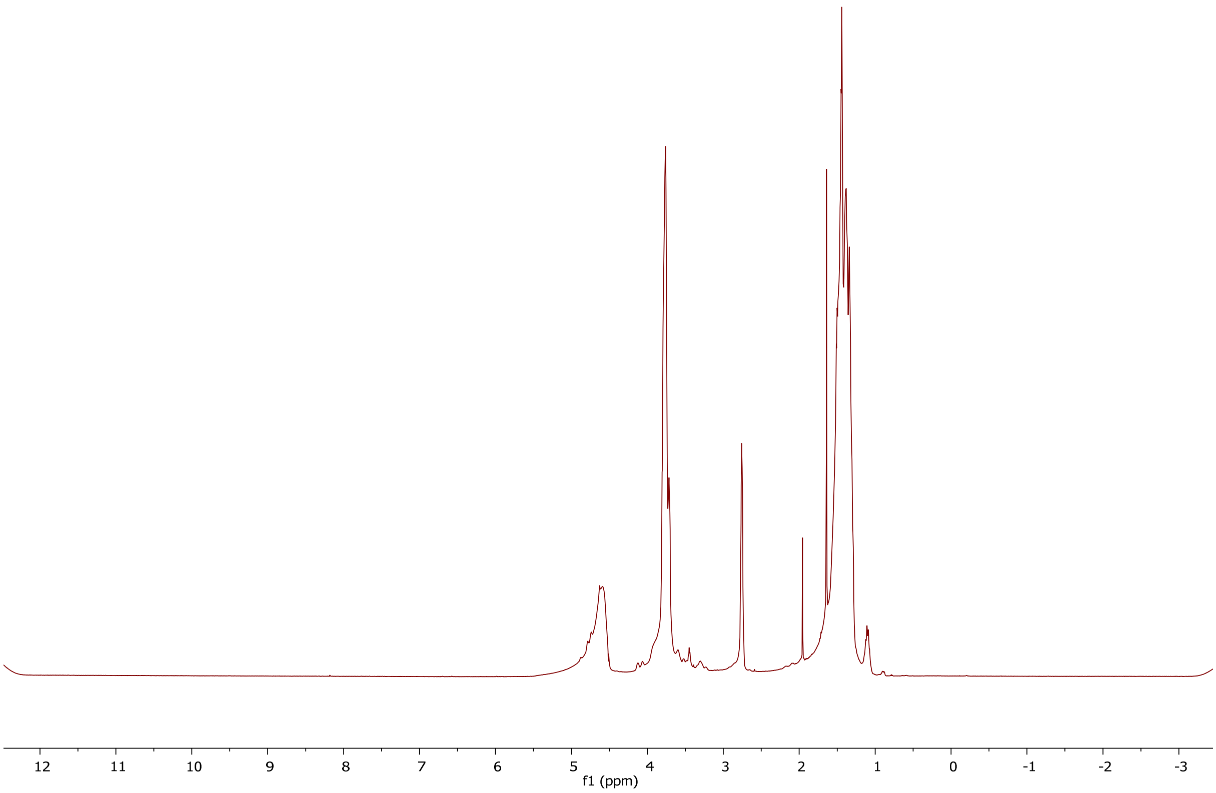

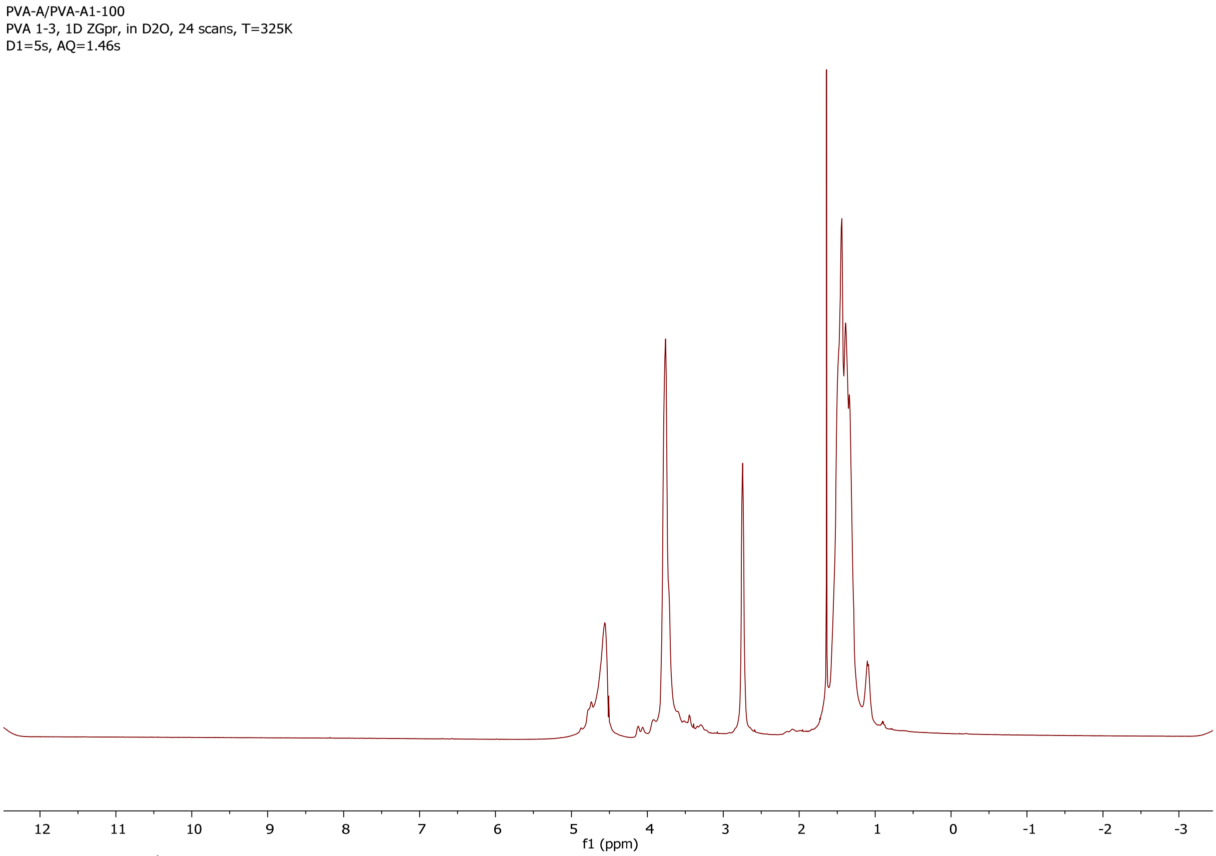

Figure S5. ^{1}H NMRs of amine modified PVA in D_2O. From top to bottom: PVA-A1-10, PVA-A1-50, PVA-A1-100

PVA-A/PVA-A2-10
PVA 2-1, 1D ZGpr, in D2O 24 scans, T=325K
D1=5s, AQ=1.46s

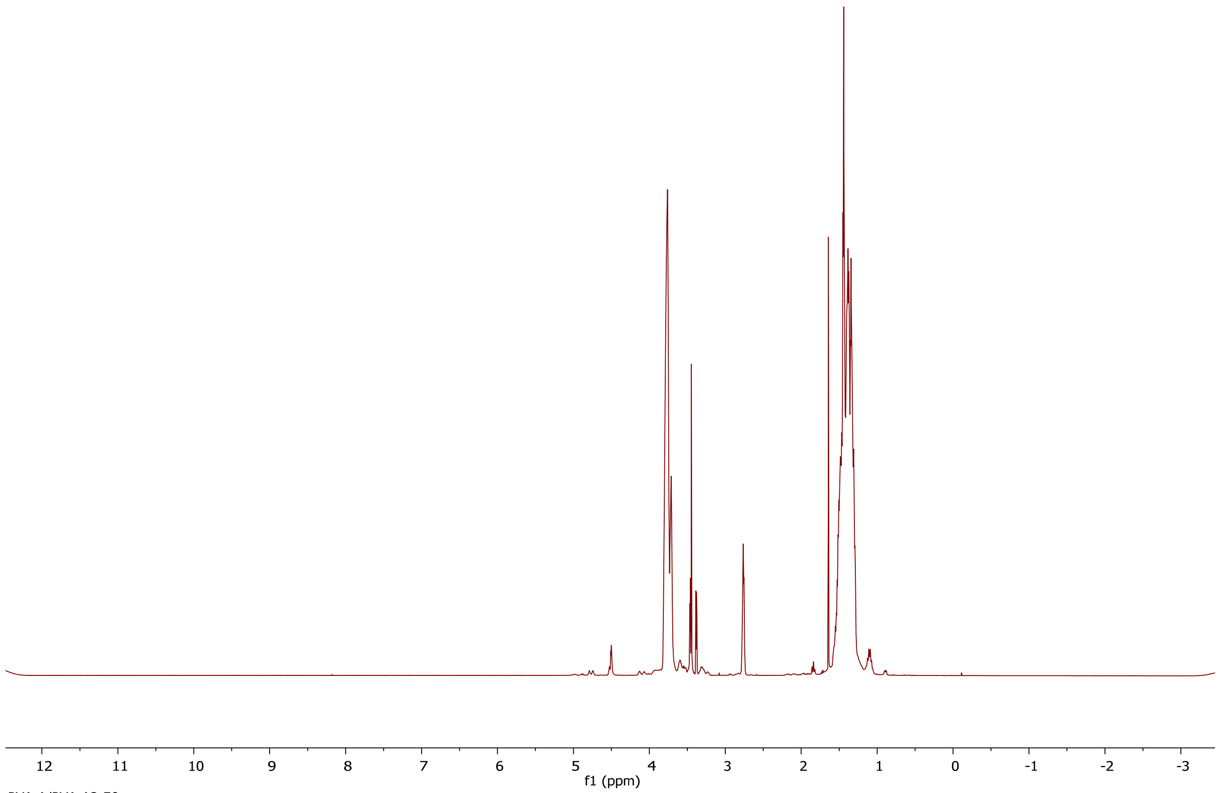

PVA-A/PVA-A2-50
PVA 2-2, 1D ZGpr, in D2O, 24 scans, T=325K
D1=5s, AQ=1.46s

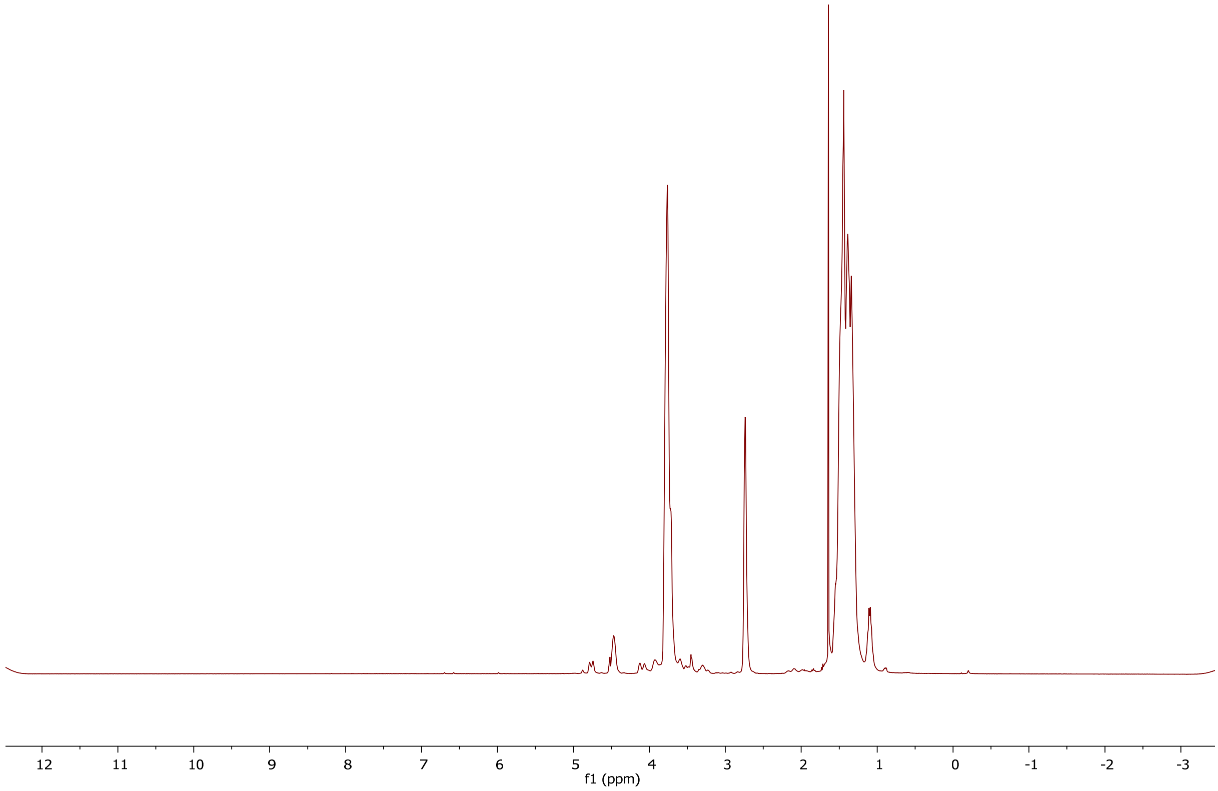

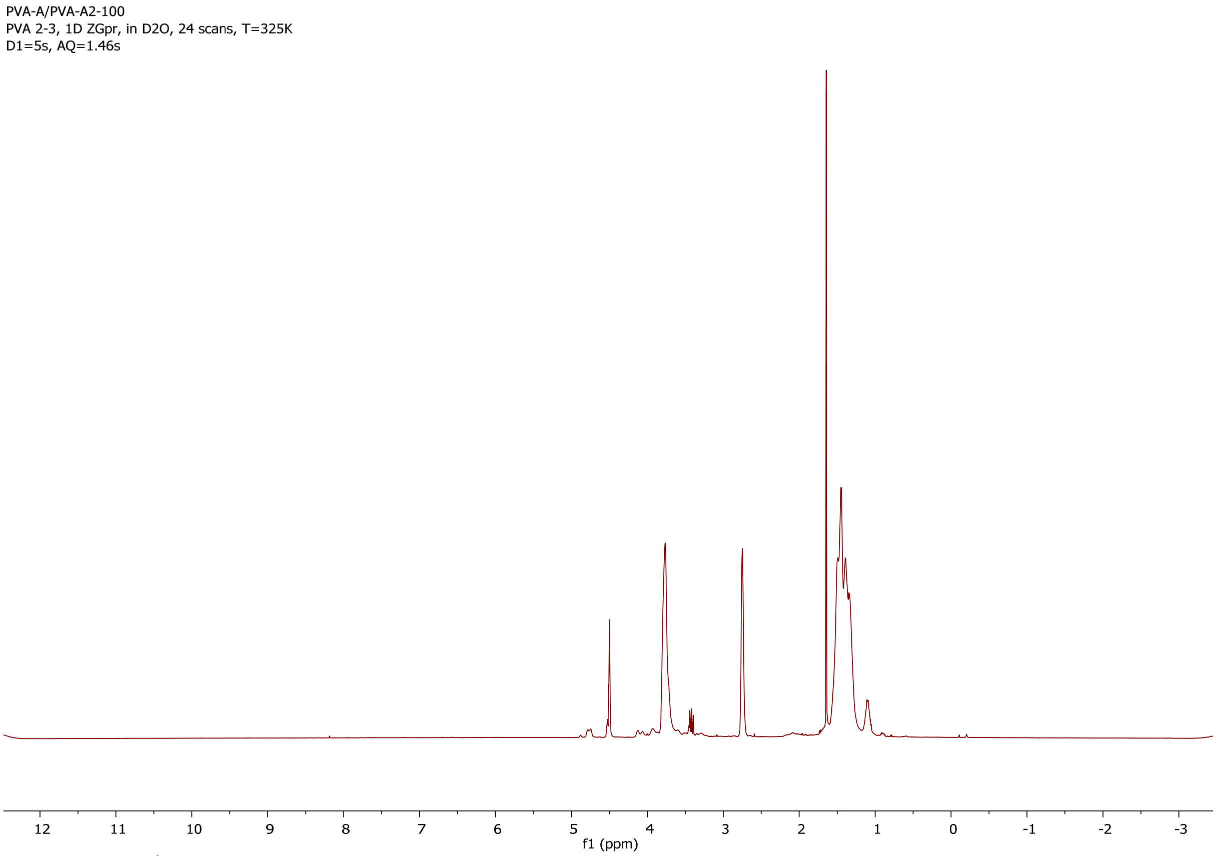

Figure S6. ^{1}H NMRs of amine modified PVA in D_2O. From top to bottom: PVA-A2-10, PVA-A2-50, PVA-A2-100,

PVA-A/PVA-A24-10
PVA 3-1, 1D ZGpr, in D2O, 24 scans, T=325K
D1=5s, AQ=1.46s

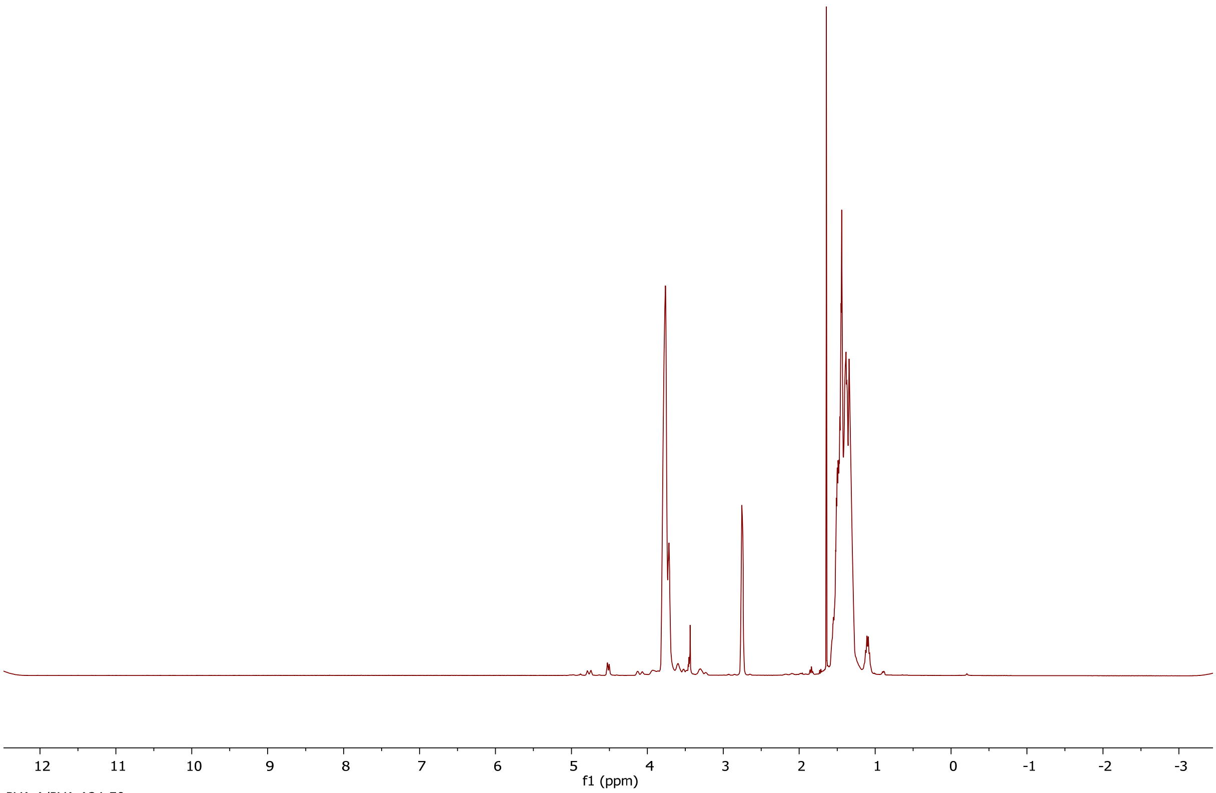

PVA-A/PVA-A24-50
PVA 3-2, 1D ZGpr, in D2O, 24 scans, T=325K
D1=5s, AQ=1.46s

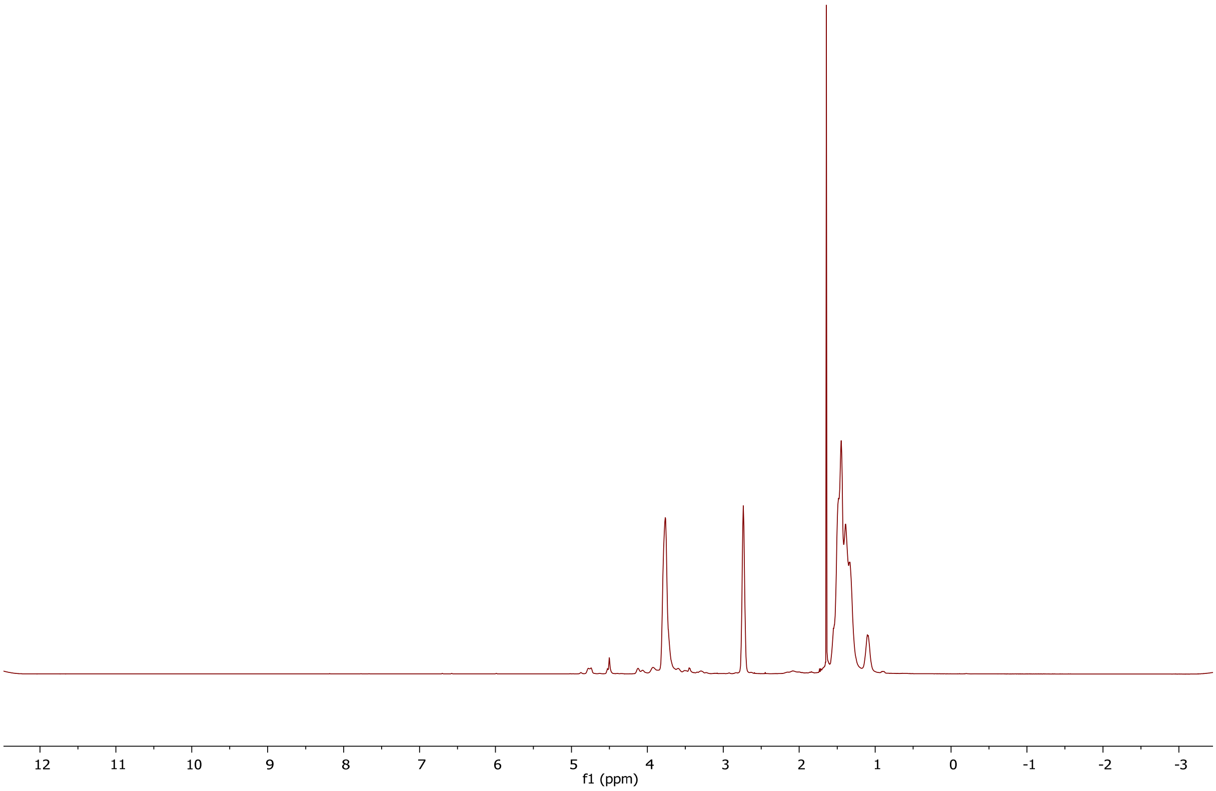

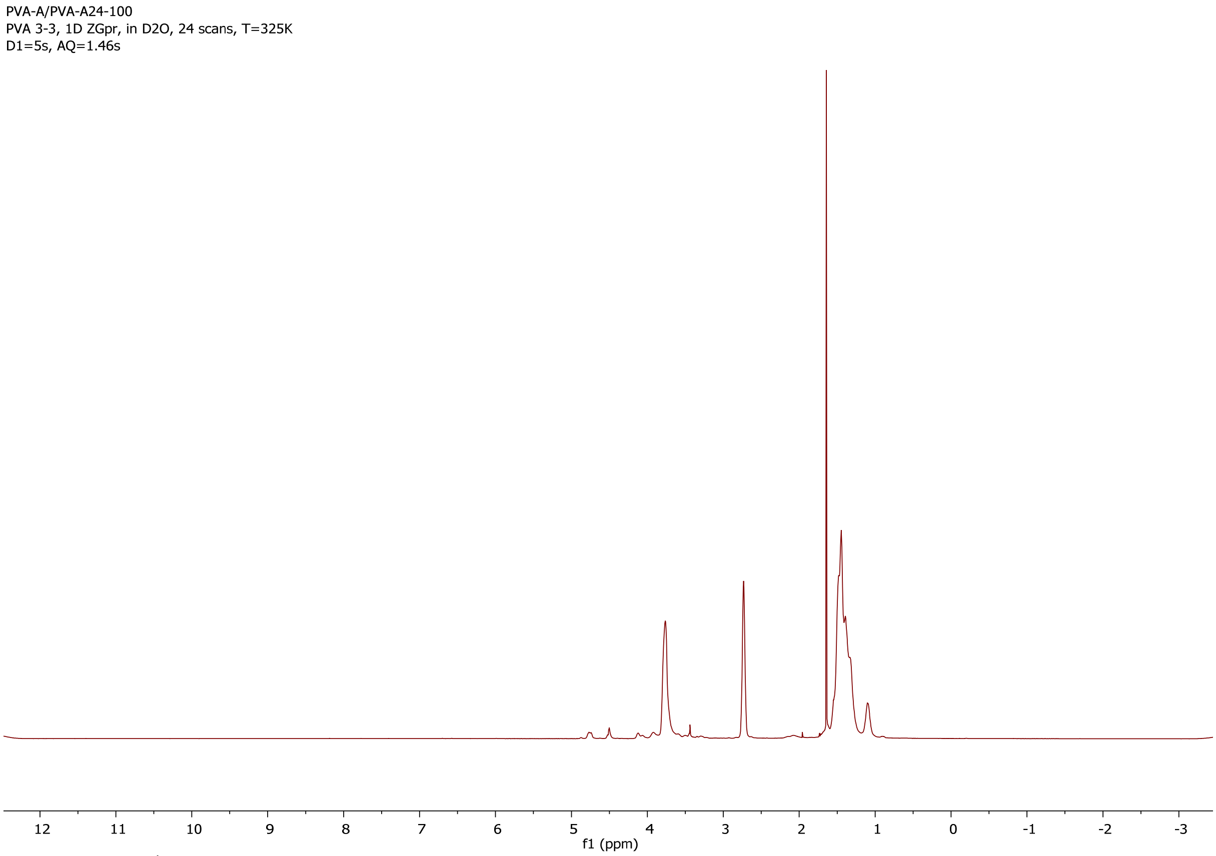

Figure S7. ^{1}H NMRs of amine modified PVA in D_2O. From top to bottom: PVA-A24-10, PVA-A24-50, PVA-A24-100

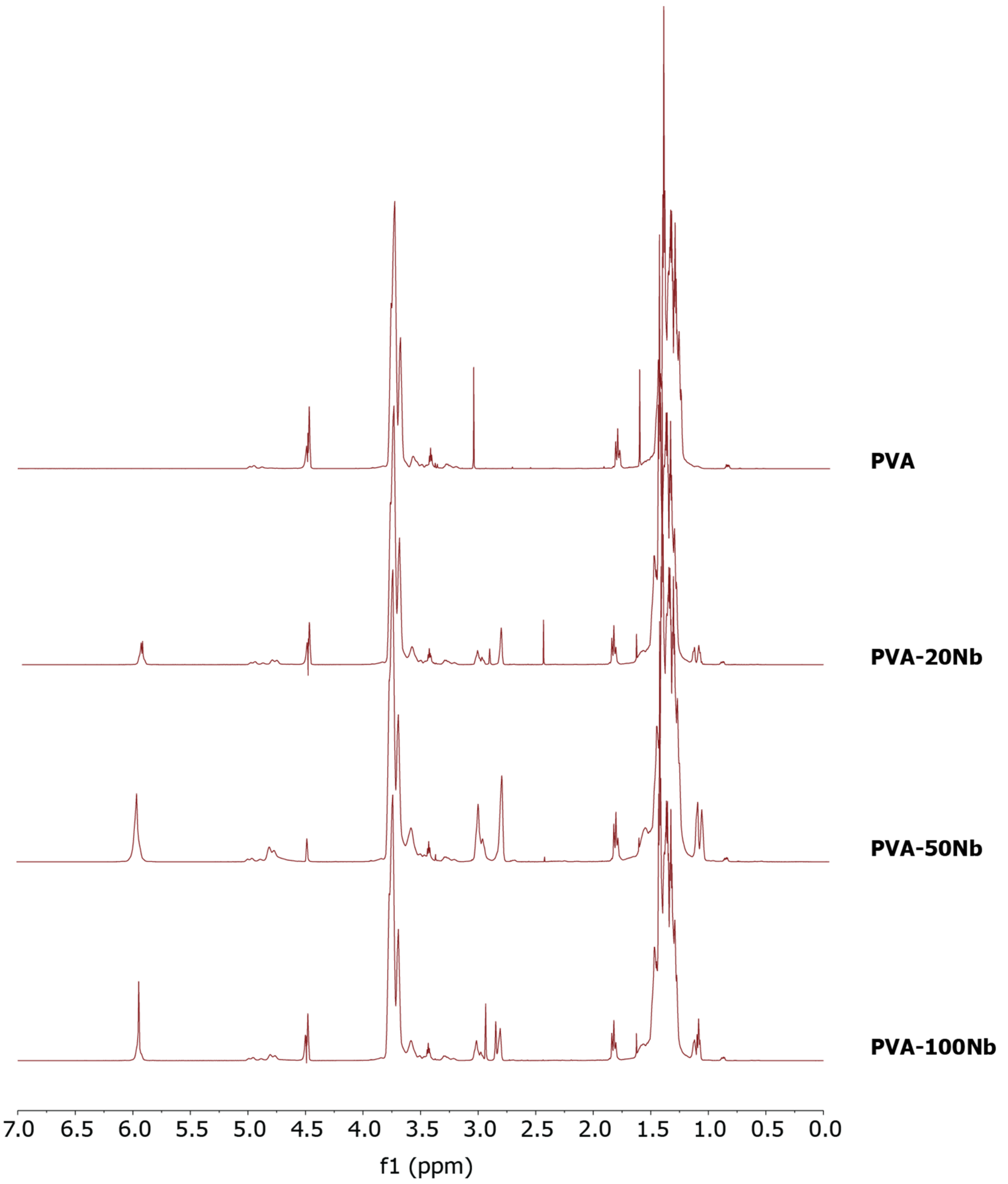

Figure S8. ^{1}H NMRs of norbornene modified PVA in D_2O. From top to bottom: PVA, PVA-Nb (0.2 equiv), PVA-Nb (0.5 equiv), PVA-Nb (1 equiv)
